# Prevalence of mental health problems among rural adolescents in India: A systematic review and meta-analysis

**DOI:** 10.1038/s41598-022-19731-2

**Published:** 2022-10-04

**Authors:** Eslavath Rajkumar, Grace Jacob Julia, N. V. Sri Lakshmi K., P. K. Ranjana, Mahesh Manjima, Rajanala Ruchitha Devi, Dubey Rukmini, George Christina, John Romate, Joshua George Allen, John Abraham, Anieta Merin Jacob

**Affiliations:** 1 Department of Psychology, Central University of Karnataka, Kalaburagi, Karnataka India; 2 Humanities & Applied Sciences, Indian Institute of Management Ranchi, Ranchi, Jharkhand India; 3 Department of Family Medicine/Geriatrics, St. John’s Medical College, Academy of Medical Science, Bangalore, Karnataka India; 4 Department of Oral Medicine and Radiology, Sri Venkateshwara Dental College and Hospital, Bangalore, Karnataka India

**Keywords:** Psychology, Risk factors

## Abstract

Adolescent mental health problems have been recognised as a major issue in low-income countries including India. Through a meta-analytic approach, the present review delineate the overall prevalence of each of the most discussed mental health problems among rural adolescents in India, comprising depression, anxiety disorders, generalised anxiety disorder, panic disorder, separation anxiety, social anxiety disorder, suicidality, hyperactivity, emotional problems, conduct problems and peer problems. The review also presents the potential determinants of such mental health problems. Using PRISMA guidelines, a total of thirty-five studies were finalized from databases such as PubMed, Science Direct, JSTOR, Web of Science, Google Scholar and ProQuest. From the findings, it is observed that male and female adolescents does not differ significantly in the prevalence of most mental health problems. However, social anxiety was found to be more prevalent among females when compared to males. In meta-regression, factors like tools used (screening tools vs diagnostic interviews), sample size, setting (school-based vs community-based), sampling technique and year of publication were found to influence the prevalence rates of certain mental health problems, reported in the studies. Major determinants influencing the prevalence of mental health problems in rural adolescents were age, socio-economic status, academic and family environment. Individual factors such as social media usage, physical activity, and substance use also contribute to mental health problems. As India accounts for one-fifth of the world's adolescent population, the findings of this review can have global implications.

## Introduction

‘Psychiatric epidemiology’ studies the causes and prevalence of mental health problems in society^1^. Ever since the establishment of the Diagnostic and Statistical Manual of Mental Disorders (DSM)^2^, numerous psychiatric epidemiological studies have been conducted across the world^3^. Due to a lack of awareness about mental health disorders in countries like India, those struggling with psychiatric issues remain unnoticed and neglected^4^. Psychiatric epidemiological studies address this issue by identifying people with psychiatric problems and by finding new occurrences of a particular mental health problem^1^.

World Health Organization (WHO) defines adolescents as those in the 10–19 age range^5^. Most unidentified mental health problems develop during adolescence phase^6,7^, many of which are life-long disorders^8^. Empirical evidence emphasises that adolescents are more vulnerable to recurrent anxiety, depression, mood disorders, and cognitive and behavioural issues as they grow up^9,10^. Mental health problems account for 45% of the burden and dysfunctionalities in the adolescent population^11,12^. If left unnoticed and untreated, such mental problems become more complicated with the transition of adolescents into adulthood. The most serious consequence of concern is a suicide, which has been a major predictor of the rising number of adolescent deaths^13,14^.

Adolescent mental health problems have been recognised as a major issue in low-income countries including India^15,16^. As per the 2011 Census, 21% of the Indian population consists of adolescents, with an estimated count of 253 million. However, there exists an incongruency between the needs and services for mental issues of adolescents in India^17–19^. The current Indian adolescent health initiatives do not acknowledge adolescent mental health with due importance. Lack of understanding about mental health problems in the country leads to adolescents experiencing their needs as incapacitated^20^.

Compared to western countries, epidemiological studies done on adolescent mental health problems in India can be found less in number^21^. Of the epidemiological studies done in India on adolescents, a wide variation in the prevalence of mental health problems from 2 to 63% can be noted^22,23^. However, inadequate description of the case, non-uniform diagnostic methods, type of area and variation in the set of mental health problems under consideration, use of non-representative samples, small sample size and discontinuation of participants after screening were some of the limitations that could be seen across the studies. The healthcare infrastructure, insufficient resources, stigma towards mental illnesses and sociocultural aspects of India limit the generalisability of the research findings conducted in other developed countries^16,19^. A meta-analysis helps systematically synthesise the results of many studies to give an overall estimate^24^. The present meta-analysis aims to provide an exact figure on the prevalence of the major mental health problems reported among adolescents in India. India has the largest number of adolescents, comprising one-fifth of the world's adolescent population^16,25^. Thus, the results of the present meta-analysis can have global implications.

The majority of the adolescent population in India resides in rural areas^26^. Previous studies have claimed that rural adolescents have more mental health issues than urban adolescents^15,16,23,27^. This can be attributed to the different lifestyles and environmental factors of the two areas^28^. Adolescents living in rural areas are neglected and exploited. They can be considered more vulnerable to mental health problems for many reasons, including their lack of access to health facilities, illiteracy, sudden exposure to metro-cities, supernatural beliefs, high prevalence of substance abuse, less preventive health screening, hostile family environments, low socioeconomic status and so on^29–31^. Venkaiah et al.,^32^ pointed out the undernutrition issue among India's rural adolescents. Poor nutritional status causes stunted growth, thus making this population more vulnerable to mental deterioration^32^. Not much-consolidated information about mental health problems of rural adolescent students could be found through literature search. As rural adolescents are a vulnerable group with many unique stressors, there is an urgency for more reliable information regarding the mental health status of rural adolescents, which will contribute to planning the necessary resources as well as preventive and control strategies for this group.

Socially constructed characteristics of women and men help understand the concept of ‘Gender’^33^. It may be a factor influencing one’s mental health^10^ as researches have shown that gender affects one's control over the determinants of socioeconomic status, access to various resources, health and many other essential aspects of life^34^. It was claimed in certain studies that the majority of the mental health problems other than emotional issues are more prevalent in boys than girls^16^; while in some studies, rural adolescent girls were found to be more mentally affected^35^ owing to the high frequency of early marriage and child-bearing in rural India, lack of autonomy, becoming a victim of marital violence, dropping out from education which in turn influence future employment, decreased social mobility and so on^36–41^. Reasonable cross-cultural differences exist regarding the prevalence of many mental disorders^42^. Contemporary research initiatives should address the lack of national statistics on mental health of adolescents^43^. The present meta-analysis gives wider clarity regarding such claims by analysing whether the prevalence of each mental health problem under study varies across gender (and other variables) or not.

The present study is the first meta-analysis in the literature that comprehensively reviews the prevalence of mental health problems among rural adolescents in India published across the 1990–2021 time period. The present study has considered a larger spectrum of mental health problems experienced by rural adolescents using selected studies that had used appropriate diagnostic measures. The following were the primary objectives:

1. To analyse the pooled prevalence of each of the mental health problems among rural adolescents in India, including depression, anxiety disorders (AD), Generalised Anxiety Disorder (GAD), panic disorder (PD), separation anxiety (SeA), social anxiety disorder (SoA), suicidality, hyperactivity, emotional problems, conduct problems and peer problems.
2. To explore variation (if any) in the prevalence of the investigated mental health problems among rural adolescents with respect to gender, year of publication, sample size, study setting, sampling technique and tools used.
3. To systematically review the included studies to identify the determinants of the investigated mental health problems among rural adolescents in India.

## Methods

### Inclusion criteria

Quantitative studies published between 1990 and 2021 were selected for pooling. Studies conducted among rural adolescents in India were included, in which the sample participants were aged between 10–19 years. In order to be included, the studies should have reported the prevalence of at least one of the following mental health problems: depression, anxiety disorders, general anxiety disorder, panic disorder, separation anxiety, social anxiety, suicidality, hyperactivity, emotional problems, conduct disorders and peer problems. Studies having adequate information to calculate the prevalence of these mental health problems, were also included.

The studies conducted on both urban and rural settings were also included if the study mentioned separate results for rural participants regarding their mental health problems; In the case of rural–urban combined results, prevalence estimates were taken if more than 60% of the participants were from the rural area. Only studies published in the English language were considered.

### Exclusion criteria

Studies with no full text available were excluded. Studies that did not use standardised tools/measurement scales or a structured psychiatric diagnostic interview, as part of the survey methodology were excluded. Studies in particular settings such as psychiatric wards in hospitals and studies conducted following any particular natural disaster etc., were excluded. Studies with ambiguous methodology or results were excluded. Conference abstracts, reviews, case studies, letters, commentaries, conceptual papers, editorials and books were excluded.

### Information sources and search strategy

A systematic search of the databases such as PubMed, Science Direct, JSTOR, Web of Science, ProQuest and Google Scholar, was conducted to identify relevant studies. The search was completed by the end of 2021, collecting all english-language publications since 1990. The search strategy was decided based on a preliminary recursive search of research databases by the first, second and third authors, which was later validated by the rest of the authors.

The following search strategy was applied: (Adolescent OR Teenager OR Adolescents OR Youth OR Adolescence OR rural adolescents) AND (India OR rural India) AND (“mental health problems" OR Anxiety OR depression OR "generalised anxiety disorder" OR "panic disorder" OR "social anxiety disorder" OR suicidality OR hyperactivity OR “mental health”) AND (Prevalence OR rate OR incidence).

The reviewers then expanded the search by identifying further studies from the references of the screened full-texts. In addition, the reviewers ran a manual search in other organisational websites to identify potentially eligible studies that could not be retrieved from the database searches. Because of the small number of papers that met the eligibility criteria during the first search, an additional search was conducted. Before final analysis, the search strategy was re-run to check for missed records (if any) that met the inclusion criteria. The search strategy used in each of the databases have been uploaded as supplementary file (“supplementary information”).

### Study selection and data extraction

The search findings were downloaded into Zotero software (Corporation for Digital Scholarship, Virginia, USA), and duplicate references were removed. The remaining studies were analysed following preferred reporting items for systematic reviews and meta-Analyses (PRISMA) guidelines^44^. Primary screening was done by checking the title and abstract of each study to identify and select the studies which met the inclusion and exclusion criteria. Subsequently, a secondary screening was done where the full text of articles was read and assessed by each reviewer independently based on the eligibility criteria. Reasons for exclusion were noted down at every stage.

After finalising the articles, relevant information like study characteristics (first author’s name, design of the study, year of publication, location of the study, sample size, instruments or tools used), sampling technique used, setting of the study (school-based or community-based), participant characteristics (age, community- rural or urban, gender) and outcomes (mental health problems and their prevalence rates) were retrieved. If a particular study selected did not  directly report results using prevalence, then it was computed using the available information. Information regarding determinants of mental health problems among rural adolescents in India was collected for the purpose of systematic review. The collected data was entered into Google sheets. The disagreements and doubts about studies were cleared through group discussion.

### Quality assessment

Two of the reviewers assessed the quality of the studies independently using the JBI critical appraisal checklist for prevalence studies^45^, published by the Joanna Briggs Institute in 2017. The evaluation criteria were: (1) appropriateness of the sample frame; (2) appropriateness of recruitment procedure; (3) adequacy of the sample size; (4) sufficiency in description of subjects and setting; (5) coverage of the identified sample; (6) validity of the methods used to identify the mental health problems; (7) reliability of the methods used to identify mental health problems symptoms or disorders; (8) adequacy of statistical analyses; and (9) appropriateness in management of response rate. Each item was assessed by scoring in the following way: yes = 1; no, unclear or not applicable = 0. Disagreements between the two authors, about the qualitative evaluation of studies were settled by seeking validation from other authors, complemented by the calculation of the inter-judge agreement index. Total quality scores of less than 5, 5 to 7 and greater than 7 were interpreted as low, moderate and high quality, respectively.

### Data analysis

Pooled prevalence rates of mental health problems were calculated in the meta-analyses using the random-effects model, weighted by the inverse of the variance. The pooled prevalence was reported with 95% confidence intervals: Lower Limit − Upper Limit (CI: LL − UL). The "metaprop" command of the Stata software (version 17.0) was used for the analyses.

Heterogeneity was assessed by the I^2^ statistic, which describes the percentage of variation across studies due to heterogeneity rather than chance^46^. It is a ratio that ranges between 0 and 100%^47^. The higher the I^2^ value/ percentage, the greater the heterogeneity between the studies. Values of 25%, 50% and 75% represented cut-off points for low, moderate and high degrees of heterogeneity, respectively. Results were considered to be statistically significant only if p value was less than or equal to 0.05.

Subgroup analyses stratified by gender, year of publication, sample size, setting (school-based vs community-based), sampling method (probability sampling vs non-probability sampling) and tools used (screening tool vs diagnostic interview) were conducted to investigate potential sources of heterogeneity between subgroups. Subgroup differences were tested by meta-regression analysis (STATA V.17.0 ‘metareg’ command).

Besides a visual inspection of the funnel plot, potential publication bias was assessed through Egger’s test (checking the small study effects), with the results indicating publication bias when p value was less than or equal to 0.05. Sensitivity analysis was also done to confirm the consistency of prevalence estimates.

A systematic review to identify the determinants of the investigated mental health problems in rural adolescents in India was completed through a narrative synthesis of the included studies.

## Results

### Search flow and study characteristics

The initial search identified 2776 records through database searching using the mentioned keywords. On removal of 1214 duplicates, 1562 articles were selected for title/abstract reading. Consequently, 128 articles were selected based on inclusion criteria, and the remaining 1448 articles were excluded. Full texts of 2 articles could not be retrieved.

After a full-text screening of retrieved articles, 96 articles were removed based on reasons such as unavailability of full-texts, lack of differentiation between urban and rural adolescents, absence of prevalence rates mentioned and having prevalence estimates indicating combined results of children and adolescents. Studies with a special population were also removed, i.e., patients in psychiatric hospitals or survivors of natural disasters as participants. Duplicates and review papers were also removed. By the end of the full-text screening, 30 studies were included.

**Figure 1:**
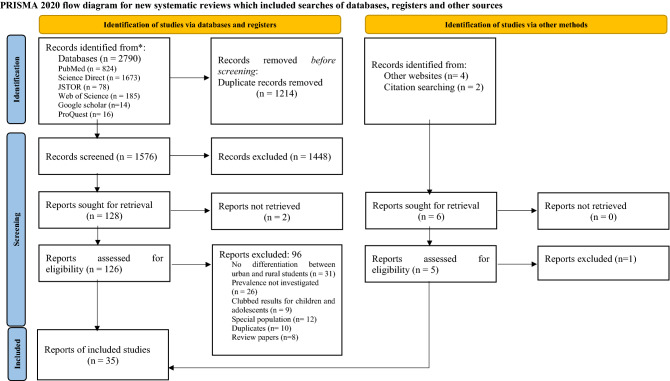
PRISMA flowchart detailing the study selection process.

Five more studies which met the inclusion criteria were added in the final stage through a manual search of other websites, complemented by searching for citations from included studies. Thus, 35 studies were selected for the qualitative synthesis (systematic review), and 34 studies were included for quantitative synthesis (meta-analysis). Figure 1 shows the PRISMA flowchart depicting the study selection process.

The 35 studies provided prevalence data for common mental health problems for 17,514 participants. The participants were adolescents between the ages of 10–19 years. Five studies reported prevalence estimates for female adolescents and three reported prevalence for male adolescents only. The remaining 27 studies mentioned the prevalence estimates for both males and females (combined or separately). There were 13 studies done in South India (Karnataka (6), Kerala (4), Tamil Nadu, Puducherry, Andhra Pradesh) and 22 studies done in North India (Haryana (5), Maharashtra (2), Jharkhand (4) Uttar Pradesh (2), Jammu& Kashmir, Gujarat (2), Punjab, West Bengal (2), Rajasthan, Chandigarh, Assam).

Out of the 35 studies, 24 were school-based and the remaining 11 were community-based. Fifteen studies used probability sampling, while 20 studies used non-probability sampling methods.

All studies that reported the prevalence of emotional, conduct, and peer problems had used the Strengths and Difficulties Questionnaire (SDQ) for screening. The tools used to screen all the remaining mental health problems varied across the selected studies.

The articles were all cross-sectional studies, and were conducted between the years 1990–2021 in various rural areas within India. Table 1 depicts the characteristics of the 35 studies in detail.

**Table 1:** Study characteristics (n = 35).

Sl. No.	First author	Year of publication	Study setting	Location of the study	Variables	Tools	Sample size	Quality evaluation
1	Dhoundiyal	2009	Community-based study	Karnataka	emotional problems, conduct problems, hyperactivity, peer problems	SDQ	120	7
2	Samanta	2012	School-based study	West Bengal	Loneliness, suicidality	GSHS	95	6
3	Nair	2013	Community-based study	Kerala	Anxiety Disorders (AD), Panic Disorder (PD), GAD, Separation Anxiety (SeA), Social Anxiety (SoA)	SCARED, K-SADS-PL	500	8
4	Russell	2013	Community-based study	Kerala	Anxiety Disorder (AD), Depressive Disorder (DD)	SCARED, BDI, K-SADS-PL	500	8
5	Russell	2013	Community-based study	Kerala	Anxiety Disorder (AD)	SCARED	500	8
6	Kumar	2013	School-based study	Jharkhand	Suicide, depression, anxiety	SIQ, GHQ-12, HADS	79	7
7	Waghachavare	2015	School-based study	Maharashtra	Social Phobia and Depression	DASS-21, SPIN	997	9
8	Vashist	2014	School-based study	Haryana	Depression	SCL-80	945	9
9	Ahir	2015	School-based study	Rajasthan	Loneliness, suicidality	GSHS	120	7
10	Kharod	2015	School-based study	Gujarat	emotional problems, conduct problems, hyperactivity, peer problems	SDQ	966	9
11	Ali	2016	School-based study	Jharkhand	Emotional, conduct and peer problems, hyperactivity	SDQ	780	8
12	Chakraborty	2016	School-based study	Karnataka	Depression	PHQ-A	284	8
13	Bahl	2016	School-based study	Jammu	Depression, suicidality	Semi open-ended questionnaire	236	8
14	Prabha	2017	School-based study	Andhra Pradesh	Anxiety, depression	HADS	289	7
15	Manuel	2016	School-based study	Kerala	Anxiety, Panic Disorder (PD), GAD, Separation Anxiety (SeA), Social Anxiety (SoA)	SCARED	250	6
16	Satyanarayana	2017	School-based study	Karnataka	AD, depression, suicidality, hyperactivity	MINI KID	200	8
17	Nair	2017	School-based study	Gujarat	Emotional problems, conduct problems, hyperactivity, peer problems	SDQ, TSQ	264	7
18	Shukla	2019	School-based study	Uttar Pradesh	Depression	KADS	336	9
19	Singh	2017	School-based study	Chandigarh	Depression	PHQ-9	68	8
20	Archana	2017	Community-based study	Karnataka	Social Anxiety	SPIN	446	8
21	Yadav	2017	School-based study	Jharkhand	Suicide	APS	500	7
22	Shaikh	2018	School-based study	Maharashtra	Depression, Anxiety	DASS-21	461	9
23	Mishra	2018	Community-based study	Uttar Pradesh	Depression, Anxiety disorder (AD)	CDI, RCMAS	100	7
24	Yuvaraj	2018	Community-based study	Puducherry	Social Phobia	SPIN	1018	9
25	Madasu	2019	Community-based study	Haryana	AD, GAD, PD, SeA, SoA	SCARED, MINI KID	678	9
26	Rai	2019	School-based study	Punjab	Loneliness, suicidality	GSHS	100	6
27	Beattie	2019	Community-based study	Karnataka	Depression, Suicidality	Samata baseline survey tool for girls Cohort 2	1191	8
28	Edlina	2019	School-based study	Assam	Emotional problems, conduct problems, hyperactivity, peer problems	SDQ	437	9
29	Hanspal	2019	School-based study	Karnataka	Depression	PHQ-9	223	9
30	Rose-Clarke	2020	Community-based study	Jharkhand	Suicidality	BPC	3324	8
31	Chopra	2020	School-based study	Haryana	Depression	CDI	250	7
32	Dwivedi	2020	School-based study	West Bengal	Emotional problems, conduct problems, hyperactivity, peer problems	SDQ	184	9
33	Mohta	2020	Community-based study	Haryana	Depression	PHQ-9, MINI KID, MINI	583	9
34	Priyanga	2020	School-based study	Tamil Nadu	Separation Anxiety (SeA), Social Anxiety (SoA), Panic Disorder (PD), Generalised Anxiety Disorder (GAD)	SCARED	500	8
35	Rajamani	2021	School-based study	Haryana	Social Phobia	SPIN	120	6

*GSHS*-Global School-based Student Health Survey, *SCARED*-Screen for Child Anxiety Related Emotional Disorders, *K-SADS-PL*-Schedule for Affective Disorders and Schizophrenia for School-Age Children/Present and Life-time Version, *BDI*-Beck Depression Inventory, *DASS-21*-Depression Anxiety and Stress Scale-21, *SPIN*-Social Phobia Inventory, *SDQ*-Strengths and Difficulties Questionnaire, *PHQ-A*-Patient Health Questionnaire-9 modified for Adolescents, *MHS-CONNERS-3*-Multi Health Systems (MHS)- CONNERS-3, *HADS*-The Hospital Anxiety and Depression Scale, *MINI KID*-Mini International Neuropsychiatric Interview for children/adolescent, *TSQ* -Teenage Screening Questionnaire-Trivandrum, *KADS*-Adolescent Depression Scale, *PHQ-9*-Patient Health Questionnaire, *BPC*-Brief Problem Checklist, *CDI*-Children’s Depression Inventory, *RCMAS*-Revised Children’s Manifest Anxiety Scale, *MINI*-Mini International Neuropsychiatric Interview, *IPAQ*-International Physical Activity Questionnaire, *AD*-Anxiety Disorders, *PD*-Panic Disorders, *SoA*-Social Anxiety, *SeA*-Separation Anxiety, *GAD*-General Anxiety Disorder, *DD*-Depressive Disorder, *ADHD*-Attention Deficit Hyperactivity Disorder, *MDD*-Major Depressive Disorder.

### Quality of studies and publication bias

Using JBI quality appraisal criteria, all included studies were evaluated for their quality. Twelve studies were found to have moderate risk of bias/moderate quality^9,16,20,35,48–55^ while the rest 23 studies had a low risk of bias/high quality^15,22,23,26–28,56–72^. The inter-judge agreement index (κ) was calculated to be 0.88, indicating a high agreement between the judges about the quality of the studies. Most studies did not meet the following three parameters: (1) appropriateness of recruitment procedure, (2) adequacy of the sample size, and (3) appropriateness in management of response rate. Many studies employed non-probability sampling techniques, which do not yield a representative sample of the target population. Very few studies provided evidence that the respective authors determined an adequate sample size and used a small sample size using sample size calculation. Details of response rate was not reported in majority of the studies.

Considering the insufficiency of data for certain mental health problems, the assessment of publication bias could be done only for depression, anxiety disorders, social anxiety and suicidality. The visual inspection of the funnel plot of studies and Egger's test revealed an absence of publication bias (p > 0.05).

### Determinants of mental health problems among rural adolescents in India

Factors such as gender, age, socio-economic status, academic environment and family environment influence the prevalence of mental health problems among rural adolescents. Individual factors such as social media usage, physical activity and substance use also affect the prevalence of mental health problems.

Table 2 summarizes the results reported on the prevalence of mental health problems among rural adolescents in India. Sensitivity analysis conducted by sequentially removing each study proved the stability in the prevalence estimate of each mental health problem.

**Table 2:** Prevalence of mental health problems among rural adolescents in India: overall estimate and subgroup analysis

	No. of studies (k)	No of participants (n)	Prevalence (%)	95% CI	I² (%)	p valueª between groups
**Depression**	17	7944	27	17–38	99.08**	
**Gender**						
Male	7	1603	29	12–50	98.53**	0.96
Female	9	3922	31	15–50	99.22**	
**Year of publication**						
2009–2015	4	2521	20	12–30	96.72**	0.44
2016–2021	13	5423	29	16–45	99.27**	
**Sample size **						
< 500	11	2310	31	17–48	98.47**	0.34
≥ 500	6	5634	9	7–36	99.51**	
**Setting**						
School-based	13	5572	34	23–45	98.58**	0.05*
Community-based	4	2372	9	5–13	98.94**	
**Sampling**						
Probability sampling	10	4962	26	15–39	98.83**	0.86
Non-probability sampling	7	2982	28	11–49	99.27**	
**Tool used**						
Screening tool	13	6561	34	23–47	99.05**	0.04*
Diagnostic interview	4	1383	8	3–14	92.09**	
**Anxiety Disorders (AD)**	9	3057	26	16–37	97.82**	
**Gender**						
Male	8	1234	25	11–41	97.20**	0.85
Female	8	1773	28	17–40	96.42**	
**Year of publication**						
2009–2015	2	579	26	22–30	–	0.98
2016–2021	7	2478	25	13–40	98.36**	
**Sample size **						
< 500	6	1379	30	14–47	97.76**	0.29
≥ 500	3	1678	19	13–25	91.01**	
**Setting**						
School-based	6	1779	30	15–48	98.32**	0.33
Community-based	3	12778	19	12–26	89.04**	
**Sampling**						
Probability sampling	3	857	18	12–25	70.68*	0.36
Non-probability sampling	6	2200	29	16–45	98.35**	
**Tool used**						
Screening tool	6	2079	31	17–47	98.25**	0.19
Diagnostic interview	3	978	16	14–19	0.00	
**Generalized Anxiety Disorder (GAD)**	4	1928	16	7–28	97.73**	
**Panic Disorder (PD)**	4	1928	20	6–41	98.99**	
**Separation Anxiety (SeA)**	4	1928	18	1–47	99.50**	
**Social Anxiety Disorder (SoA)**	8	4509	23	17–30	96.53**	
**Gender**						
Male	7	2161	16	11–22	90.46**	0.05*
Female	8	2348	28	20–37	95.46**	
**Year of publication**						
2009–2015	2	1497	20	18–22	–	0.59
2016–2021	6	3012	25	15–35	97.49**	
**Sample size **						
< 500	3	816	37	34–41	2.34	0.00**
≥ 500	5	3693	17	12–22	93.88**	
**Setting**						
School-based	4	1867	23	12–36	96.92**	0.90
Community-based	4	2642	24	15–34	96.83*	
**Sampling**						
Probability sampling	4	3139	23	15–33	96.95**	0.95
Non-probability sampling	4	1370	23	11–38	96.93**	
**Suicidality**	9	5798	9	5–13	95.27**	
**Year of publication**						
2009–2015	3	294	17	5–35	91.88**	0.17
2016–2021	6	5504	6	3–10	95.42**	
**Sample size **						
< 500	6	830	10	5–18	89.43**	0.55
≥ 500	3	4968	7	2–14	98.05**	
**Setting**						
School-based	7	1330	11	6–18	90.45**	0.04*
Community-based	2	4468	4	4–5	–	
**Sampling**						
Probability sampling	3	815	18	8–30	92.95**	0.02*
Non-probability sampling	6	4983	5	3–8	86.71**	
**Hyperactivity**	7	2951	7	2–14	97.15**	
**Year of publication**						
2009–2015	2	1086	21	19–24	–	0.00**
2016–2021	5	1865	4	3–6	53.42	
**Sample size **						
< 500	5	1205	5	3–8	72.57	0.26
≥ 500	2	1746	13	11–15	–	
**Sampling**						
Probability sampling	2	621	6	4–8	–	0.69
Non-probability sampling	5	2330	7	1–17	98.0**	
**Emotional problems**	6	2751	9	6–13	89.90**	
**Conduct problems**	6	2751	19	11–29	97.00**	
**Peer problems**	6	2751	15	2–36	99.33**	

*CI* Confidence interval.

ªp values for meta-regression (upto two decimal places).

**p ≤ 0.01, *p ≤ 0.05.

#### Depression

Seventeen studies reported the prevalence of depression among rural adolescents in India. The pooled prevalence of depression across the 17 studies was found to be 27% (95% CI: 17–38%, I^2^ = 99.08%). The prevalence of depression varied from 3.5% to 88.3% across the studies^15,20,22,23,26,27,35,50,54,55,58–60,63,64,69,71^.

In subgroup analyses, the heterogeneity remained high. It was observed that the prevalence of depression is higher in studies that used screening tools compared to diagnostic tools (34% vs 8%) and in school-based studies than in community-based studies (34% vs 9%) and in studies with a sample size of less than 500, compared with those with sample size equal to or greater than 500 (31% vs 9%).

The meta-regression analysis found no significant between-group difference for gender, year of publication, sample size and sampling (all p > 0.05). Significant between-group difference was found for tools used (p = 0.04) and setting (p = 0.05).

#### Anxiety disorders (AD)

Nine studies reported the prevalence of anxiety disorders among rural adolescents in India. The pooled prevalence of anxiety disorders was found to be 26% (95% CI: 16–37%, I^2^ = 97.82%). The prevalence of anxiety disorders ranged from 12.4% to 60% across the nine studies^20,22,26,35,50,51,56,67,72^.

In subgroup analyses, the heterogeneity remained high, and it was observed that the prevalence of anxiety disorders is higher in studies that used screening tools compared to diagnostic tools (31% vs 16%) and in studies that used non-probability sampling than those that used probability sampling (29% vs 18%). The prevalence was higher in school-based studies than community-based studies (30% vs 19%) and in studies with a sample size of less than 500, compared with those with a sample size equal to or greater than 500 (30% vs 19%).

In the meta-regression analysis, no significant between-group difference was found for gender, year of publication, sample size, setting, sampling and tool used (all p > 0.05).

#### Generalised anxiety disorder (GAD)

Four studies reported the prevalence of generalised anxiety disorder, which ranged from 7.2% to 37.2% across the studies^51,56,67,72^. The pooled prevalence of generalised anxiety disorder was found to be 16% (95% CI: 7–28%, I^2^ = 97.73%). Subgroup analyses, meta-regression, and tests for publication bias were not performed for generalised anxiety disorder due to the small number of studies and insufficient data.

#### Panic disorder (PD)

Four studies reported the prevalence of the panic disorder, which ranged from 6.6% to 55.2% across the studies^51,56,67,72^. The pooled prevalence of panic disorder could be seen as 20% (95% CI: 6–41%, I^2^ = 98.99%). Due to the small number of studies and inadequate data, subgroup analyses, meta-regression and tests for publication bias were not performed for panic disorder.

#### Separation anxiety (SeA)

Four studies reported the prevalence of separation anxiety disorder, which ranged from 2.1% to 74% across the studies^51,56,67,72^. The pooled prevalence of separation anxiety was found to be 18% (95% CI: 1–47%, I^2^ = 99.50%). Subgroup analyses, meta-regression and tests for publication bias were not performed for separation anxiety because of inadequate data and small number of studies.

#### Social anxiety disorder (SoA)

The pooled prevalence of social anxiety disorder was found to be 23% (95% CI: 17–30%, I^2^ = 96.53%). The prevalence ranged from 8.4% to 39.7% across the eight studies^51,55,56,59,65–67,72^.

In subgroup analyses, the heterogeneity remained high. It was observed that the social anxiety disorder prevalence is higher among females than males (28% vs 16%) and in studies with a sample size of less than 500, compared with those with a sample size equal to or greater than 500 (37% vs 17%).

No significant between-group difference was found for year of publication, setting, and sampling (p > 0.05) in the meta-regression analysis. Significant between-group difference was found only for gender (p = 0.05) and sample size (p = 0.00).

#### Suicidality

Across the nine studies investigating suicidality, the pooled prevalence was 9% (95% CI: 5–13%, I^2^ = 95.27%). The prevalence ranged from 2.1% to 34.2% across the studies^9,15,22,49,50,52,53,64^.

In subgroup analyses, the heterogeneity remained high, and it was observed that the prevalence of suicidality is higher in studies published between 2009 and 2015 compared to those published between 2016 and 2021 (17% vs 6%) and in studies that used probability sampling than that used non-probability sampling (18% vs 5%). However, this might be a result of the study by Kumar et al.,^50^ that reported a much greater prevalence of suicidality than all the other included studies.

In the meta-regression analysis, no significant between-group difference was found for the year of publication and sample size (p > 0.05). Significant between-group difference was observed only for setting (p = 0.04) and sampling method (p = 0.02).

#### Hyperactivity

The pooled prevalence of hyperactivity was found to be 7% (95% CI: 2–14%, I^2^ = 97.15%). Seven studies investigated hyperactivity and the prevalence ranged from 2% to 22.7% across the studies^16,22,28,48,61,62,68^.

The heterogeneity remained high, and it was observed that the prevalence of hyperactivity is higher in studies published between 2009 and 2015 compared to those published between 2016 and 2021 (21% vs 4%) in the sub-group analysis. In the meta-regression analysis, no significant between-group difference was found for sample size and sampling (p > 0.05). However, a significant between-group difference was observed only for the year of publication (p = 0.00).

#### Emotional, conduct and peer problems

Six studies reported the prevalence of emotional, conduct, and peer problems. The pooled prevalence of emotional problems, conduct problems and peer problems was found to be 9% (95% CI: 6–13%, I^2^ = 89.90%), 19% (95% CI: 11–29%, I^2^ = 97.00%), and 15% (95% CI: 2–36%, I^2^ = 97.00%) respectively. Across the six studies, the prevalence of emotional problems, conduct problems and peer problems ranged from 5.12% to 20%, 7.06% to 53.3% and 6% to 45.9% respectively^16,28,48,61,62,68^. Due to the small number of studies and inadequate data, subgroup analyses, meta-regression and tests for publication bias were not performed for emotional, conduct and peer problems.

## Discussion

The adolescent period is marked by reliance on friends more than family for mental support. Adolescents can be encouraged to spend more time and effort with friends to prevent them from experiencing issues of peer problems, conduct problems and other consequent emotional troubles^16^. Since the finalised studies in this review incorporated participants from various regions of India, the overall population of 17,514 can be regarded as an equitable representation of rural adolescents in India. The rates of morbidity, malnutrition and mortality reported in the rural areas of India are higher than what is being reported among the Indian population at large^73^. The present analysis shows that many rural adolescents in India are affected by mental health problems. Given the dearth of government-funded mental health treatments currently available, prevalence estimates for the mental health problems examined in this study highlight the need for enhanced mental health care^74^.

The high prevalence of depression found through meta-analysis re-validates the claims made by Steptoe et al., (2007) asserting Asian countries to have high levels of depression. It was interesting to see that, unlike the other types of anxiety disorders, social anxiety disorder was more prevalent among females when compared to males. As reported by previous empirical findings, female vulnerability to social anxiety might have genetic determinants besides the environmental aspects. The pooled prevalence of hyperactivity in the present study is higher than the worldwide prevalence of ADHD for adolescents, i.e., 5.29^75^. This suggests that adolescents in India's rural areas need to pay greater attention to the problem of hyperactivity. The prevalence of suicidality reported in the present meta-analysis is higher when compared to the estimates reported by the latest meta-analysis on suicidal behaviour among adolescents (rural and urban taken together) in India, which found the pooled prevalence of suicidal ideation in a lifetime as 5.458%^76^. In addition, the pooled prevalence estimates for suicidality reported in the present study is also higher than that reported across other Asian countries^77^.

The high heterogeneity between the individual studies could be due to the variation in sample size, sampling techniques, tools used and the study settings. Variables like tools used (screening tools vs diagnostic interviews), sample size, setting (school-based vs community-based) and year of publication were found to influence the prevalence rates reported in the studies.

Gender-wise comparison made in some studies reveal that females are more likely than males to experience depression, emotional issues, anxiety disorders, generalised anxiety disorder, panic disorder, social anxiety, and separation anxiety^16,20,28,35,51,56,59,63,67,72^. Males were found to have more hyperactivity, conduct and peer problems than females^16,20^. But some other studies found no significant association of gender with depression and/or anxiety disorders^26,35,50,57,71^. However, the present meta-analysis shows gender to have a significant association with social anxiety disorder only and not with other mental health problems.

The prevalence of depression, generalised anxiety disorder, panic disorder and suicidality increased with age^56,57,60,63,69–71^. There were mixed results regarding the relationship of age with anxiety disorders, emotional problems, conduct problems and peer problems respectively^16,20,26,35,56,61^. Social anxiety was found to be more prevalent in early and middle adolescence^65,66^.

Several comorbid conditions were also identified in the rural adolescents. The common psychiatric comorbidities with depression were found to be anxiety disorders, panic disorder, generalised anxiety disorder, separation anxiety^58^, conduct disorder in the past year, oppositional defiant disorder (ODD) in the past six months, attention deficit hyperactivity disorder (ADHD) in the past six months^71^, suicidal ideation^23,49,55,71^ and social anxiety^59^. Adolescent boys with anxiety and depressive disorder were identified to be in the high-risk group for suicidal behaviour^47^.

Students of government schools had a greater prevalence of depression. However, a lesser prevalence of anxiety disorders was observed in students from government schools than those from private schools^23,56,60^. In a study by Shukla et al., (2017), it was interesting to notice that adolescent girls studying in Hindi medium schools were about 12 times more preponderate for depression^26^.

Family factors such as a report of parental discord^23,69^, harsh parenting and strained family relationships^26^ were significantly associated with higher depression and anxiety rates. Sharing a living room with siblings, death of a family member in the recent past, presence of a family member with serious medical or psychiatric illness^71^, alcoholism or substance abuse in the family^23^, financial constraints and altercations in the family due to it^23^, were some of the identified determinants of depression among rural adolescents. Also, adolescents in joint families had a significantly higher prevalence of depression and social anxiety^20,65^. The majority of the studies reported a higher prevalence of depression and anxiety disorders in adolescents belonging to families of lower socioeconomic status^20,22,23,60,67,69^.

Depression was significantly higher in adolescents with illiterate parents and in adolescents whose parents were unemployed^27^, while no association was found between parents' occupation status and anxiety disorder in the child^51^. Feeling pressurised by parents to perform well in exams^69^, lack of supportive environment in school, spending lesser number of hours studying and lack of participation in cultural activities and/or sports in school^23,64^ were some factors associated with higher levels of depression, emotional, peer and conduct problems in adolescents. Prevalence of anxiety and depression was higher among students with unsatisfactory academic performance^23,26^. After-school entertainment like watching movies and spending time with friends had a favourable effect on mental health^16^. Also, individual resilience, supportive family and school environments and yoga were identified as protective factors^23,65,71^.

Adolescents smoking cigarettes or chewing tobacco and those who had experienced adverse events in the recent past, had significantly higher depression and anxiety^26^. Spending more time on social media sites was related to lesser depression and having a romantic partner^23^ was found to be linked to higher levels of depression. Adolescents with a long-term medical illness and/or impaired body image were more vulnerable to having depression^71^.

Mixed results were observed for the association of physical activity with emotional, conduct and peer problems respectively. However, hyperactivity was found to be less prevalent among adolescents with sedentary behaviour^16,28,61^.

The present review highlights the need for screening, early identification, monitoring and treatment of mental health problems. Improving awareness and coordination among parents, teachers and community workers can significantly help achieve this^9,20,59,60,68,69^. Some studies emphasized the development of psychosocial care, targeted interventions and mental health education programs for adolescents^15,23,62,63^. Life skill education was suggested to deal with mental health issues^16,61^. Yoga, sports and other recreational activities must be encouraged to improve mental well-being^28^. Present meta-analysis results emphasise the importance of giving vigilant supervision and careful attention to rural adolescents to prevent them from getting involved in anti-social behaviours (drug abuse, stealing and so on) and attempting suicide^2,78^.

## De-limitations, limitations and scope for future research

Though the heterogeneity value across studies was high for most mental health problems, subgroup analysis investigating the reason for heterogeneity could not be explored for some mental health problems due to the insufficiency of empirical studies that met the inclusion criteria. For a few mental health problems for which subgroup analyses were done, variables like gender (male/female), year of publication (2009–2015/2016-2021), sample size (< 500/≥ 500), setting (school-based/community-based), sampling (probability vs non-probability sampling) and tools used (screening tool/diagnostic interview) could not account for the heterogeneity. However, considering the commonality of these limitations in other similar meta-analysis studies^76,79^, less variability in the pooled prevalence estimate level, and consistency with similar prevalence estimates reported by other meta-analysis results on particular mental health problems, interpretability of the present review's prevalence rates can be considered as valid.

The majority of the mental health problems were found to be identified using different screening methods, which would have caused variation in the prevalence estimate range obtained across the finalized studies. This, in turn, would have affected the present meta-analysis results. In addition, certain studies have used self-report measures to identify the prevalence of certain mental health problems. When disorders are self-reported, it sometimes becomes impossible to identify the isolated, sub-threshold, mild or short-lived cases that can be diagnosed and cured in the early stages. The authors of multiple studies mentioned that the 'social desirability' factor could have impacted the subject's responses, especially when questions were asked about substance abuse or the family and home atmosphere. Hence it would have influenced the accuracy of results reported in such studies.

The samples selected for some of the finalised studies focused majorly on specific regions or towns and did not provide a true representation of that particular study's population. Therefore, more empirical studies should provide results for a wider geographical area by taking larger samples that gives more generalisable and practical findings.

Since all of the included studies had used cross-sectional designs, inferences regarding the lifetime prevalence of each mental health problem could not be made in the present review. Therefore, there exists a scope for more research on this aspect of the topic. In addition to this, most of the included studies were school-based studies. As school-based studies are known for higher prevalence than community studies, this could have influenced the overall prevalence rates reported in the present review.

The search for appropriate literature was limited to only English indexed papers and journals. Access to certain published articles could not be gained as they were unavailable online. Despite all the limitations, the fact that there is a dire need to build, focus and implement psychological care services in rural India stays undisputed.

## Implications

Finding the prevalence of mental health problems in the adolescent population of rural India aids in planning and distributing the nation's mental health resources appropriately. The study results would encourage public health planners, private health sectors, educational sectors and parents to take active measures to address adolescent mental health issues. This is very important as adolescence is the most vulnerable, yet developing stage of life. Timely addressing and management of affected rural adolescents can potentially smoothen their transition from adolescence to adulthood, hence, helping to adapt better to the complexities and consequences they may face in later phases of life. This study emphasises the importance of implementing appropriate primary mental health care services in the country's rural areas and the proves the necessity to include mental health awareness in education policies. Present meta-analysis results contribute to monitoring the effectiveness of new mental health policies and programs. High prevalence rates of mental health problems in rural adolescents of the country point out the urgency for reforming the public health policies, thereby facilitating more appropriate interventions and quality counselling services to be put in place for the better mental health of future adults. This, in turn, helps the development of the rural areas too.

Also, the study suggests placing teaching staff or trained professionals who can identify potential behaviours in students, that may lead to psychological disturbances later. Such students can be sent to experts in the field to be diagnosed and given adequate support at a very preliminary stage itself. Considering all the given points, it is not wrong to say that finding the prevalence of mental health issues is an indirect way to assess the success of mental health reform in the country. 

## Conclusion

Though meta-analysis is to be conducted once every 10 years, there has not been a meta-analysis that comprehensively reviews the prevalence of mental health problems among rural adolescents in India for the past 30 years. The current review provides accurate prevalence estimates for a range of mental health problems that affect adolescents in rural areas. The findings show that adolescents in rural India have a significant incidence of mental health problems. Therefore, the Indian government must expand and enhance the current national mental health services to cater to the requirements of adolescents living in rural areas of the nation.

## Supplementary Information

Supplementary Information.

## Data Availability

The datasets generated during and/or analysed during the current review paper, are available from the corresponding author on reasonable request.
