# Supplementary material for: Prevalence of mental health problems among rural adolescents in India: A systematic review and meta-analysis
Source: Sci Rep. 2022 Oct 4;12:16573. doi: 10.1038/s41598-022-19731-2 (PMC9532445; doi:10.1038/s41598-022-19731-2)
Supplement: Supplementary file 1 — Supplementary Information. [file 41598_2022_19731_MOESM1_ESM.pdf]

SUPPLEMENTARY FILE-1 (Search Strategy used)

Databases	Search strategy	Articles identified
**PUBMED**	((((Adolescence OR youth OR adolescents OR rural adolescents)) AND ((prevalence OR rate))) AND ((Mental health problems[Title/Abstract] OR Depression[Title/Abstract] OR anxiety[Title/Abstract] OR suicidality[Title/Abstract] OR generalized anxiety Disorder[Title/Abstract] OR panic disorder[Title/Abstract] OR separation anxiety[Title/Abstract] OR social anxiety[Title/Abstract] OR hyperactivity[Title/Abstract] OR mental health[Title/Abstract]))) AND ((India[Title/Abstract] OR rural India[Title/Abstract])) Filters: Clinical Trial, Randomized Controlled Trial, Clinical Trial, Randomized Controlled Trial, from 1990/1/1 - 2021/12/31	824
**WEB OF SCIENCE**	(((TS=(Adolescence OR adolescents)) AND TS=(Mental health problems OR Depression OR anxiety OR suicidality OR generalized anxiety Disorder OR panic disorder OR separation anxiety OR social anxiety OR hyperactivity OR mental health)) AND TS=(India OR rural India)) AND TS=(prevalence OR rate)	185
**JSTOR**	((((Adolescence OR adolescent OR rural adolescents) AND (Mental health problems OR anxiety OR depression OR suicidality OR hyperactivity)) AND ab:(India OR rural India)) AND (Prevalence)) AND la:(eng OR en)	78
**SCIENCE DIRECT**	(Adolescents OR rural adolescents) AND (Mental health problems OR Depression OR anxiety OR suicidality OR hyperactivity) AND (India) AND (prevalence)	1673
**PROQUEST**	AB, TI(Adolescence OR adolescents OR rural adolescents) AND (Mental health problems OR Depression OR anxiety OR suicidality OR generalized anxiety Disorder OR panic disorder OR separation anxiety OR social anxiety OR hyperactivity OR mental health) AND AB, TI("India" OR rural India) AND (prevalence OR rate)	16

OTHER ONLINE RESOURCES USED FOR MANUAL SEARCH

- Google Search
- National Digital Library of India
- Research Gate
- Shodhganga
- WorldWideScience.org
